# Consolidating Clinical Insights and Uncovering Novel Regulatory Mechanisms of Exosomal MicroRNAs in Obesity and Metabolic Dysfunction Associated Steatotic Liver Disease: A Systematic Review and Bioinformatics Analysis

**DOI:** 10.1002/fsn3.71901

**Published:** 2026-05-22

**Authors:** Qigui Mo, Amirreza Ghafourian, Masoomeh Hamdi, Arian Alidadipour, Mahdieh Soleimani, Maryam Davoudi, Xiaolei Miao, Reza Afrisham, Molood Bagherieh

**Affiliations:** ^1^ Hubei Key Laboratory of Diabetes and Angiopathy, School of Pharmacy, Xianning Medical College Hubei University of Science and Technology Xianning P. R. China; ^2^ Department of Medical Laboratory Sciences, School of Allied Medical Sciences Tehran University of Medical Sciences Tehran Iran; ^3^ Students' Scientific Research Center Tehran University of Medical Sciences Tehran Iran; ^4^ Mazandaran University of Medical Sciences Ramsar Iran; ^5^ Department of Biochemistry and Genetics, Faculty of Medicine Mazandaran University of Medical Sciences Sari Iran

**Keywords:** computational biology, exosomes, metabolic dysfunction associated steatotic liver disease, microRNAs, obesity, systematic review

## Abstract

Obesity and metabolic dysfunction associated steatotic liver disease (MASLD) are interrelated metabolic disorders characterized by chronic inflammation, insulin resistance, and dyslipidemia. While both conditions are well recognized clinically, the molecular mechanisms underlying their frequent coexistence remain poorly understood. Growing evidence indicates that circulatory exosomal microRNAs (miRNAs) act as critical mediators of inter‐organ communication. In this PROSPERO‐registered systematic review (CRD420251017335), we integrated clinical evidence with bioinformatics analyses to clarify shared miRNA‐mediated regulatory networks between obesity and MASLD. Literature was retrieved from MEDLINE, ISI Web of Science, and Embase. Bioinformatics analysis using miRWalk also revealed shared miRNAs, predicted target genes, and elucidated enriched pathways using Gene Ontology and KEGG. Clinical studies identified 93 obesity‐associated and 24 MASLD‐associated exosomal miRNAs, with *let‐7b* and *miR‐335‐5p* emerging as common nodes. Bioinformatics analysis using miRWalk revealed extensive overlap at the functional level, including 40,410 shared miRNA‐mRNA interactions and 42 common target genes. Several circulating exosomal miRNAs from obese individuals, including *miR‐122*, *miR‐192*, *miR‐128*, and *miR‐9‐5p*, were consistently associated with liver histopathology, inflammatory markers, and liver enzyme levels. Importantly, obesity‐derived *miR‐298*, *miR‐342*, and *let‐7d‐5p* showed strong diagnostic performance (AUC ≥ 0.85), exceeding that of alanine aminotransferase (ALT). Furthermore, *ADIPOQ* emerged as a central therapeutic target within the shared miRNA network, regulated by both *let‐7b* and *miR‐335‐5p*, providing a molecular link between adipose tissue dysfunction and hepatic metabolic regulation. This integrative analysis supports a unified model in which exosomal miRNAs serve as key molecular intermediaries connecting obesity and MASLD.

## Graphical abstract

Clinical studies identified 93 obesity‐associated and 24 NAFLD‐associated exosomal miRNAs, with *let‐7b* and *miR‐335‐5p* emerging as common nodes. Bioinformatics analysis revealed extensive overlap at the functional level, including 40,410 shared miRNA‐mRNA interactions and 42 common target genes. Several circulating exosomal miRNAs from obese individuals, including *miR‐122*, *miR‐192*, *miR‐128*, and *miR‐9‐5p*, were consistently associated with liver histopathology, inflammatory markers, and liver enzyme levels. *ADIPOQ* emerged as a central therapeutic target within the shared miRNA network, regulated by both *let‐7b* and *miR‐335‐5p*.

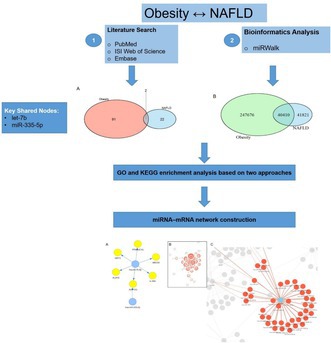

**Abbreviations**
AIH: autoimmune hepatitis
ALP: alkaline phosphatase
ALT: alanine aminotransferase
AMPK: AMP‐activated protein kinase
ANGPTL3: angiopoietin‐like protein 3
AST: aspartate aminotransferase
AUC: area under the curve
BMI: body mass index
BP: biological process
C/EBP: CCAAT/enhancer‐binding proteins
CC: cellular component
GGT: gamma‐glutamyl transferase
GO: Gene Ontology
HOMA‐IR: Homeostatic Model Assessment of Insulin Resistance
KEGG: Kyoto Encyclopedia of Genes and Genomes
MAFLD: Metabolic Dysfunction‐Associated Fatty Liver Disease
MAPK: mitogen‐activated protein kinase
MASLD: metabolic dysfunction associated steatotic liver disease
MetS: metabolic syndrome
MF: molecular function
miRNAs: microRNAs
NAFLD: non‐alcoholic fatty liver disease
NASH: nonalcoholic steatohepatitis
PPARγ: peroxisome proliferator‐activated receptor gamma
ROC: receiver operating characteristic
sEVs: small extracellular vesicles
T2DM: type 2 diabetes mellitus

## 1 Introduction

Overweight and obesity are defined as excessive adipose tissue accumulation, which leads to dysfunctional physiological processes or causes various health issues and adversely affects health outcomes. Regarding this condition's prevalence, statistics from the Institute for health metrics and evaluation (IHME) indicated a minimum of 3.68 million deaths attributable to obesity in 2025 (Institute for Health Metrics and Evaluation 2025). An issue that was thought to be specific to high‐income nations has now proliferated within low‐ and middle‐income regions as well (Mayoral et al. 2020). Obesity has shown significant involvement in the pathogenesis of a variety of health complications, including type 2 diabetes mellitus (T2DM), hypertension, and metabolic dysfunction associated steatotic liver disease (MASLD). Furthermore, obesity has also exhibited correlations with compromised glucose metabolism, insulin, and leptin resistance, low‐grade inflammatory states, and modifications in sympathetic nervous system activity linked to a reduction in noradrenergic neuronal function and thermogenic processes, thereby increasing the risk of enduring energy balance disorders (Luo and Lin 2021).

Among obesity‐related diseases, MASLD is delineated by the pathological buildup of lipids within hepatocytes in individuals who do not consume alcohol in significant quantities, specifically defined as less than 20 g per day for males and under 10 g per day for females (Mavrogiannaki and Migdalis 2013). Being connected with metabolic syndromes, particularly type 2 diabetes mellitus and visceral fat accumulation, this hepatic disease's significance within the continuum of insulin resistance and associated metabolic dysregulation is emphasized (Anstee et al. 2013). As indicated by global statistics, MASLD showed a prevalence of approximately 30% in the global population (Amini‐Salehi et al. 2024). The conditions of overweight and obesity substantially contribute to metabolic diseases, including MASLD. However, certain obese individuals may be defined as metabolically healthy obese (MHO). Factors such as adipose tissue distribution, functionality of adipose tissue, and insulin resistance are critical to the pathogenesis of metabolic disorders, including MASLD. Current research proposes various mechanisms of interaction between these two conditions, including the “liver‐pancreas axis,” which implicates insulin resistance, and the “multi‐hit hypothesis,” which suggests the role of obesity‐induced inflammation in the development of non‐alcoholic fatty liver (Godoy‐Matos et al. 2020).

New research suggests that miRNAs in the bloodstream play a role in the onset and advancement of obesity (Cabiati, Guiducci, et al. 2023; Cabiati, Randazzo, et al. 2023; Ferrante et al. 2015; Santamaria‐Martos et al. 2020; Patra et al. 2024; Pan et al. 2019; Togliatto et al. 2016; Eirin et al. 2021; Li et al. 2021) and MASLD (Gim et al. 2021; Zhou et al. 2020; Jiang et al. 2021; Li et al. 2020; Newman et al. 2022; Zhang and Pan 2022; Paluschinski et al. 2023; Liu et al. 2020, 2021; Samy et al. 2024; Infante‐Menéndez et al. 2023; Kan Changez et al. 2023). Small vesicles (sEVs) located outside of cells that mediate communication between cells are known as exosomes. These sEVs carry bioactive substances such as RNA, DNA, and proteins. Within these vesicles, miRNAs function as post‐transcriptional regulators of gene expression and influence a broad spectrum of biological processes, including adipocyte differentiation, insulin sensitivity, inflammation, and lipid metabolism (Dandare et al. 2023). Some miRNAs modulate adipose tissue hypertrophy and epigenetic regulation (e.g., C/EBP), while others modulate inflammation (via PPARγ), insulin sensitivity (PPAR‐δ), or adipokine secretion (e.g., ANGPTL3), the ultimate result being ectopic fat deposition (Benavides‐Aguilar et al. 2023). In MASLD, miRNAs have been found to target key metabolic pathways such as de novo lipogenesis, fatty acid uptake, mitogen‐activated protein kinase (MAPK) signaling, autophagy, and nuclear receptor pathways (Kan Changez et al. 2023; Fang et al. 2021; Atic et al. 2023).

Importantly, several obesity‐associated miRNAs identified in adipose tissue or circulating exosomes target pathways directly relevant to hepatic metabolism. For example, *miR‐34a*, which is upregulated in adipose tissue from obese individuals and associated with increased TNFα and IL‐6 levels (Pan et al. 2019; Afrisham et al. 2025), is also consistently elevated in MASLD and NASH patients, where it correlates with ALT, AST, and disease severity (Zhou et al. 2020). This overlap suggests that obesity‐related miRNA dysregulation may prime the liver for steatosis and inflammation through systemic circulation. Interestingly, thyroid hormones might also play an essential function in MASLD; latest publications also indicate a connection between (intrahepatic) *miR‐34a‐5p* and the occurrence of hormonal disturbances, for example, obesity driven upregulation of miRNA and reduction of target genes (Alison‐Michelle Naujack et al. 2024). Moreover, this miRNA is controversy associated the progression of MASLD to liver cancer. Without intervention, MASLD might eventually lead to MASH and HCC. This aspect might also hint toward a clinical use of exosomal microRNAs as biomarker (Tobaruela‐Resola et al. 2025).

The present study attempts to elucidate the shared molecular pathways between obesity and MASLD using an integrative approach through combining a systematic review with bioinformatics. In this study, we first performed a systematic review to identify miRNAs associated with each condition. We collected and summarized the findings from selected studies to highlight recurrent miRNAs linked to metabolic dysfunction, inflammation, and liver pathology. To integrate the datasets and identify common regulatory elements, we constructed a Venn diagram that revealed miRNAs shared between obesity and MASLD. This approach allowed us to move beyond individual study results and focus on miRNAs with potential mechanistic relevance to both conditions. To further validate and expand these findings, bioinformatics analyses were conducted using publicly available miRNA databases. Shared miRNAs identified through both literature and computational approaches were then subjected to pathway enrichment analysis to explore their functional roles and regulatory networks. This analysis highlighted signaling pathways related to insulin resistance, inflammation, adipocyte function, and hepatic lipid metabolism, providing a mechanistic link between obesity and MASLD through miRNA regulation.

## 2 Methodology

### 2.1 Systematic Review

#### 2.1.1 Search Strategy

**FIGURE 1:**
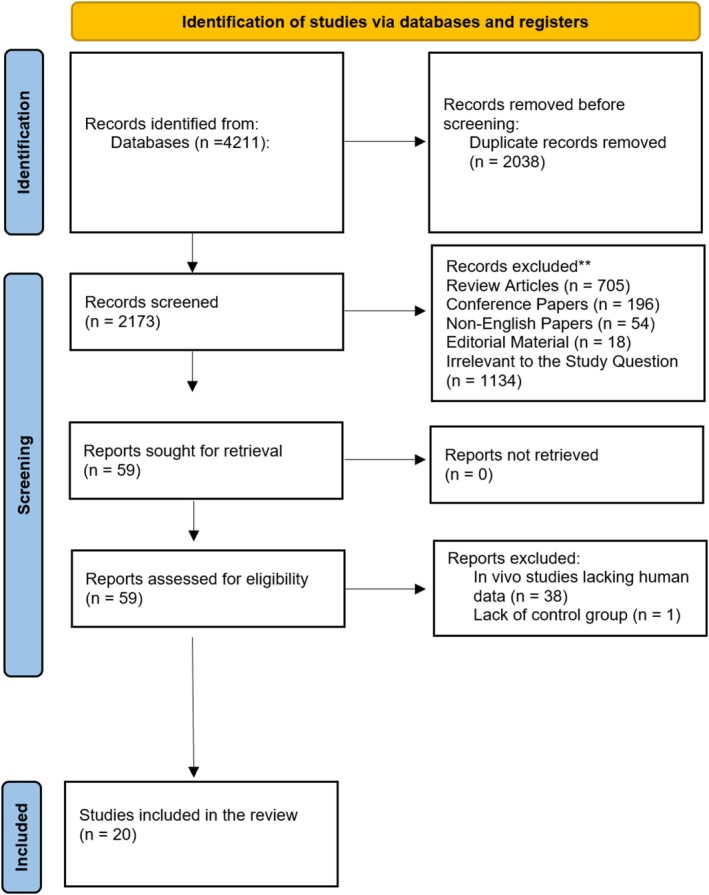
A PRISMA visual representation outlining the steps involved in selecting literature. **Irrelevant to the study question means they were not on EV miRNAs or MASLD/obesity patients.

Following the PRISMA 2020 guidelines (Figure 1) (Page et al. 2021), a systematic search was performed on September 11, 2025, across PubMed, ISI Web of Science, and Scopus without time restrictions. MeSH and non‐MeSH terms were used to identify studies on exosomal miRNAs in obesity and MASLD (Supporting Information: “*Search Strategy*”). Duplicates were removed using EndNote. The applied protocol is registered at the International Prospective Register of Systematic Reviews (https://www.crd.york.ac.uk/prospero/; PROSPERO ID: CRD420251017335).

#### 2.1.2 Study Selection

Experiments were chosen according to three research questions: (i) Which are the obesity‐altered exosomal miRNAs? (ii) In what way does MASLD influence the composition of exosomal miRNAs? (iii) What are the alterations in exosomal miRNAs that occur when obesity is considered a risk factor for MASLD?

Inclusion criteria were as follows: All samples from clinical research or ex vivo studies on (a) obese patients, (b) MASLD patients, and (c) obese patients with MASLD. Studies including participants of any age were considered; no age restriction or subgrouping was applied during study selection due to heterogeneity in reporting (some studies reported mean age rather than a specific age range). Nevertheless, age was systematically extracted as part of the data collection process and is presented in the corresponding tables. Where possible, comparisons or subgroup analyses based on age were performed and reported in the Results section due to their clinical relevance. However, other potential confounders, such as sex, insulin resistance‐related parameters, ethnicity, and comorbidities, could not be evaluated due to limitations in the available data. Most of the included studies were conducted in mixed populations of men and women, and the data relevant to the objectives of this review were not reported separately by sex in the original articles. Therefore, sex‐specific comparisons were not feasible. In addition, indicators of insulin resistance, including fasting insulin levels and HOMA‐IR, were reported for NAFLD participants in only one study (Infante‐Menéndez et al. 2023). Consequently, a reliable comparison of insulin resistance‐related variables between the two conditions was not possible. Also, because the included studies for each condition were conducted in populations with different ethnic backgrounds, ethnicity was also not consistent across studies and therefore could not be reliably evaluated or compared in our analysis. Regarding comorbidities, considering all possible comorbidities would have substantially broadened the scope of the study and reduced the comparability of the two conditions. For this reason, studies including patients with other hepatic comorbidities were excluded (e.g., hepatic neoplasms, nonalcoholic steatohepatitis (NASH), or MAFLD without a separate MASLD group). Consequently, our analysis focused only on individuals with MHO, NAFLD, or those presenting both conditions. The corresponding data for these groups were extracted and reported in separate tables.

All samples from clinical research or ex vivo studies on (a) obese patients, (b) MASLD patients, and (c) obese patients with MASLD. Both randomized and nonrandomized clinical studies published in English were considered eligible. Limiting inclusion to English‐language articles may have introduced language bias and was recognized as a limitation of this review. We excluded in vivo/in vitro research, reviews, conference abstracts, non‐English‐language articles, and book chapters. In vivo studies were included if they also involved the evaluation of human samples. Title/abstract screening and full‐text assessment were performed according to these criteria.

#### 2.1.3 Data Extraction

Two reviewers independently extracted data; disagreements were resolved by supervisors. Data was extracted regarding alterations in exosomal miRNA composition (Up/Down) in patients with obesity and MASLD, and correlation of them with metabolic parameters, including BMI (AASLD Practice Guidance; Rinella et al. 2023), Systolic/Diastolic blood pressure (mmHg; EASL‐EASD‐EASO guidelines; EASL, EASD, and EASO 2024), Insulin (mIU/L) and Homeostatic Model Assessment of Insulin Resistance (HOMA‐IR) (ElSayed et al. 2025); Lipid profile (i.e., Total cholesterol (mg/dL), Triglyceride (mg/dL), HDL (mg/dL), LDL (mg/dL)) (Rinella et al. 2023; EASL, EASD, and EASO 2024), as well as Glucose (mmol/L) and HbA1c (%) (ElSayed et al. 2025).

Due to heterogeneity in EV sources, demographic data of our patients (e.g., gender and age), miRNAs, a qualitative combination of data was performed. Key findings were summarized in tables categorized by clinical and exosomal miRNA findings in obesity, in MASLD, and in both conditions. Although we extracted information on miRNA sources, given the exploratory aim of this study to comprehensively identify candidate exosomal miRNAs potentially linking obesity and MASLD, studies employing different miRNA profiling platforms (e.g., qPCR or high‐throughput sequencing) and normalization strategies were included without methodological restriction. Effect sizes and statistical powers were mentioned where appropriate. Due to methodological heterogeneity, no meta‐analyses were performed.

#### 2.1.4 Risk of Bias Assessment

**FIGURE 2:**
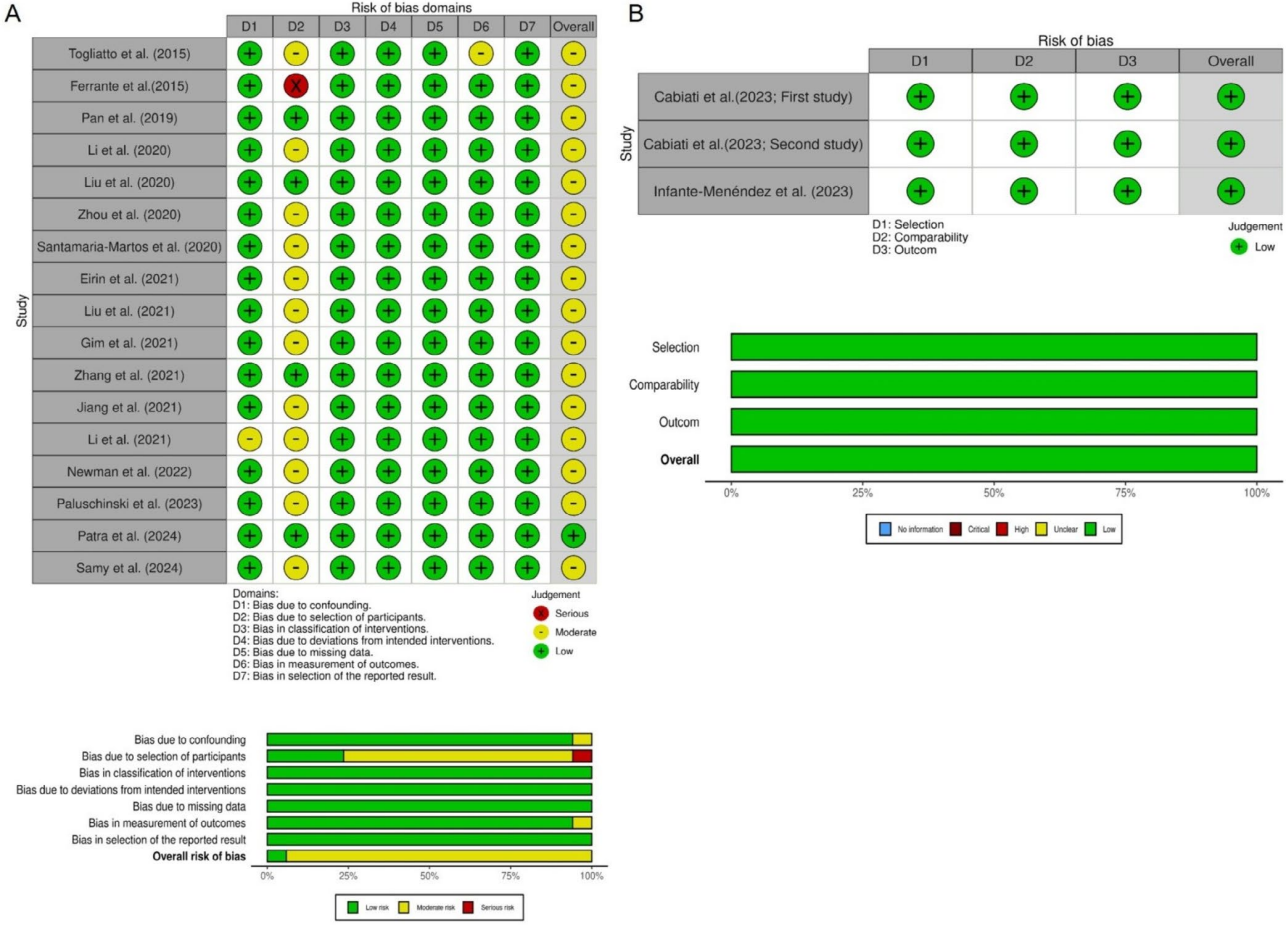
Bias assessment for (A) non‐randomized observational studies using the ROBINS‐I tool and (B) cross‐sectional studies using the Newcastle‐Ottawa Scale (NOS).

Two reviewers assessed study quality using the ROBINS‐I tool for non‐randomized observational studies and the Newcastle‐Ottawa Scale (NOS) for cross‐sectional studies. Data is presented in Figure 2.

### 2.2 Bioinformatics Analysis

To complement the systematic review, we performed a bioinformatics analysis to identify shared miRNA‐mRNA interactions and regulatory pathways between obesity and MASLD. For this study, experimentally validated interactions reported in humans were prioritized and used for interpretation of key regulatory hubs. Predicted interactions were included only if supported by consensus across multiple algorithms, with stringent filtering to reduce false positives.

#### 2.2.1 Identification of Shared miRNAs and Their Target Gene Prediction

We mined the miRWalk database (http://mirwalk.umm.uni‐heidelberg.de/) to dig deeper. miRWalk was chosen as the primary platform because it integrates multiple miRNA–target prediction tools (including TargetScan and miRDB) and also provides access to experimentally validated miRNA–mRNA interactions retrieved from miRTarBase, thereby increasing confidence in biologically relevant targets (Dweep et al. 2014). We applied disease‐specific filters to retrieve two sets of distinct miRNAs, a set of those associated with obesity, and a second set of those with MASLD. The two were compared and shown to share some (overlapping) miRNAs. Intersection was visualized using the VennDiagram R package (version 4.3.3). Subsequent analysis was conducted on those miRNAs present in both diseases. Target genes of the shared miRNAs were identified using both predicted miRNA–mRNA interactions and experimentally validated interactions available via miRTarBase within the miRWalk database.

#### 2.2.2 Functional Enrichment Analysis

Only those interactions with experimental support were carried forward for functional enrichment and network construction. To find possible functions of such target genes, Gene Ontology (GO) and Kyoto Encyclopedia of Genes and Genomes (KEGG) pathway enrichment analysis was performed. GO analysis allowed us to classify the gene functions into Biological Process (BP), Molecular Function (MF), and Cellular Component (CC). For a broader view, we conducted KEGG pathway analysis to see in which biological pathways these genes are involved. We also used the Metascape platform to integrate functional annotations from multiple resources, including GO, KEGG, Reactome, and WikiPathways. The cutoff for significantly enriched terms was as follows: adjusted *p*‐value < 0.05, minimum count ≥ 3, enrichment factor > 1.5. Results were graphed using the ggplot2 package in dot plots.

#### 2.2.3 Constructing the miRNA‐mRNA Interaction Network

The network was constructed in R using tidygraph, ggraph, and igraph packages. In the network graph, edges represented the regulatory interaction between either the target genes or miRNAs and the nodes. Node size and color were adjusted to highlight key features, such as how central a node is to the network or whether it is a miRNA or mRNA. This visualization helped us identify central regulators—miRNAs or genes that may play crucial roles in both obesity and MASLD.

## 3 Results

### 3.1 Systematic Review Summary

The initial database search yielded 4211 records. After removing 2038 duplicates, 2173 articles remained for title and abstract screening. Fifty‐nine articles were selected for full‐text review, of which 48 were excluded. A total of 20 studies met the inclusion criteria (Figure 1).

#### 3.1.1 Obesity‐Associated Exosomal miRNAs

Nine (2015–2024) studies in Italy, the USA, Spain, China, and India compared exosome miRNA profiles between obese and non‐obese individuals (Table 1) (Cabiati, Guiducci, et al. 2023; Cabiati, Randazzo, et al. 2023; Ferrante et al. 2015; Santamaria‐Martos et al. 2020; Patra et al. 2024; Pan et al. 2019; Togliatto et al. 2016; Eirin et al. 2021; Li et al. 2021). Populations examined included adolescents up to the elderly and both males and females. Exosomes were primarily isolated from plasma or serum using exoRNeasy kits, ExoQuick, ultracentrifugation, or double‐centrifugation. In the case of using adipose tissue for studies, mechanical isolation with filtration was also used.

**TABLE 1:** Summary of clinical and exosomal miRNA findings in obesity studies.

References	Year and country	Study groups	Age	Female:male	BMI (kg/m^2^)	Glucose profile	Lipid profile	Other clinical parameters	Findings	Exosome isolation (source, method)
(Cabiati, Guiducci, et al. 2023)	2023; Italy	Age‐ and sex‐matched normal‐weight (*n* = 20) and obese (*n* = 30) (in a subset of the study population) (Ssp, *n* = 23), exosome isolation was carried out: (G1) Normal weight in the subset and (G2) Obese in the subset	13.1 12.2 [subsets (G: 13.1 and G2 12.7)]	9:11 18:12 [subsets G1: 8:8 and G: 5:2]	20.2 ± 0.2 29.8 ± 0.8 [subsets G1: 20.2 ± 0.2 and G2: 30.2 ± 2.0]	FBS (mmol/L): 4.93 ± 0.09 4.3 ± 0.12 [subsets G1: 4.88 ± 0.12 and G2: 4.21 ± 0.35] HOMA‐IR: 0.95 ± 0.09 2.5 ± 0.24 [subsets G1: 0.95 ± 0.1 and G2: 2.18 ± 0.53] HbA1c (%): 5.31 ± 0.09 5.48 ± 0.06 [subsets G1: 5.26 ± 0.12 and G2: 5.51 ± 0.09]	Total cholesterol (mg/dL): 142.03 ± 8.6 170.793 ± 6.7 [subsets G1: 148.22 ± 8.75 and G2: 173.08 ± 3.82] Triglyceride (mg/dL): 73.48 ± 18.42 103.49 ± 12.49 [subsets G1: 77.58 ± 20.00 and G2: 89.09 ± 12.73] HDL (mg/dL): 47.55 ± 2.55 45.57 ± 1.41 [subsets G1: 48.89 ± 2.58 and G3: 44.15 ± 1.72] LDL (mg/dL) 81.19 ± 7.16 111.05 ± 5.37 [subsets G1: 84.23 ± 7.23 and G2: 104.44 ± 7.84]	Systolic BP (mmHg): 112.5 ± 1.8 108.0 ± 2.2 [subsets G1: 113.9 ± 1.6 and G2: 108.0 ± 4.5] Diastolic BP (mmHg): 62.2 ± 1.4 63.8 ± 1.7 [subsets G1: 62.6 ± 1.5 and G2 58.8 ± 4.9]	*miR‐33a* levels in G2: 1.33 ± 0.29 *miR‐33a* levels in G2: 0.58 ± 0.29 (*p* = 0.026) Correlation among *miR‐33a‐3p*, insulin, cholesterol, LDL, and HDL levels (*p* = 0.01)	Plasma; exoRNeasy mini/midi kit
(Cabiati, Randazzo, et al. 2023)	2023; Italy	Normal‐weight (*n* = 20) and obese (*n* = 30)	13.1 ± 0.2 12.6 ± 0.4	10:12 9:13	21.1 ± 0.6 29.5 ± 0.8	FBS (mmol/L): 4.8 ± 0.1 4.4 ± 0.1 HOMA‐IR: 1.0 ± 0.09 2.4 ± 0.3 HbA1c (%): 5.2 ± 0.07 5.4 ± 0.06	Total cholesterol (mg/dL): 145.4 ± 8.4 167.7 ± 7.4 Triglyceride (mg/dL): 81.5 ± 17.7 88.6 ± 8.7 HDL (mg/dL) 46.7 ± 2.7 45.7 ± 2.2 LDL (mg/dL) 82.3 ± 6.9 109.7 ± 6.6	Systolic BP (mmHg): 113.2 ± 1.4 112.6 ± 2.1 Diastolic BP (mmHg): 61.7 ± 1.2 65.7 ± 1.7	Decreased levels of *miR‐223‐5p* *miR‐33a‐3p* *miR‐181a‐5p*, and *miR‐199a‐5p* in the obese group	Plasma; exoRNeasy mini/midi kit
(Ferrante et al. 2015)	2015; USA	Obese (*n* = 7) and lean (*n* = 5)	15.6 ± 1.4 15.0 ± 0.7	All female	39.8 ± 2.0 23.2 ± 0.5			Increased M1 macrophage counts in obese subjects	Downregulated miRNAs: *miR‐148b* and *miR‐4269* Upregulated miRNAs: *miR‐23b* and *miR‐4429* The involvement of these miRNAs in TGF‐β and Wnt/β‐catenin signaling pathways	Visceral and subcutaneous adipose tissue ExoQuick‐TC Precipitation Solution, followed by filtration through a 200 nm microfilter
(Santamaria‐Martos et al. 2020)	2020; Spain	BMI < 30 (*n* = 19), BMI of 30–35 (*n* = 12), and BMI > 35 (*n* = 14)	45.1 ± 15.1 49.9 ± 11.3 46 ± 8.8	All female	24.8 ± 2.7 32.2 ± 1.5 38 ± 3.9	Glucose (mg/dl): 88.0 ± 9.3 92.2 ± 8.8 88.1 ± 13.4 HOMA‐IR: 0.97 ± 0.47 1.64 ± 0.75 1.74 ± 0.73 HbA1c (%): 4.59 ± 0.39 4.91 ± 0.3 4.95 ± 0.59	Total cholesterol (mg/dL): 200.7 ± 30.4 214.3 ± 50.9 208.9 ± 30 Triglyceride (mg/dL): 80.6 ± 32.1 97.5 ± 54.8 95 ± 37.8 HDL (mg/dL) 72.2 ± 14.3 60.8 ± 13.6 55 ± 9.9 LDL (mg/dL) 112.3 ± 32.1 134 ± 43.4 134.9 ± 29.4	Systolic BP (mmHg): 133.7 ± 25.7 125.3 ± 17.1 127.9 ± 8.9 Diastolic BP (mmHg): 77.2 ± 10.3 79.6 ± 7.3 78.1 ± 5.2	Exosomal *miR‐320a‐3p*: 0.1427 ± 0.1094 0.1485 ± 0.1064 0.3526 ± 0.2299 (*p* < 0.001) Exosomal *let‐7b‐5p*: 0.0271 ± 0.0278 0.0314 ± 0.0268 0.0934 ± 0.0863 (*p* = 0.001) Exosomal *miR‐186‐3p*: 0.1 ± 0.071 0.0456 ± 0.0628 0.033 ± 0.0339 (*p* = 0.004) Exosomal *miR‐106a‐5p*: 37.4 ± 29.46 75.51 ± 50.99 68.11 ± 38.51 (*p* = 0.022) Exosomal *miR‐323a‐3p*: 0.6621 ± 0.6229 1.6962 ± 1.1418 1.1332 ± 0.96 (*p* = 0.117) Exosomal *miR‐146a‐5p*: 0.2084 ± 0.1051 0.1709 ± 0.1171 0.1193 ± 0.0608 (*p* = 0.017) Exosomal *miR‐186‐3p*: 0.0114 ± 0.0087 0.0049 ± 0.0039 0.0063 ± 0.0044 (*p* = 0.046) Exosomal *miR‐106b‐5p*: 13.97 ± 14.62 21.64 ± 10.5 27.70 ± 25.4 (*p* = 0.042)	Plasma; differential centrifugation
(Patra et al. 2024)^a^	2024; India	Non‐diabetic (BMI: 18–25 kg/m^2^, FBG < 85; *n* = 16) and diabetic (BMI > 30 kg/m^2^, FBG < 120; *n* = 14)	Not mentioned	17:13	18–25 > 30				A significant increase in the level of *miR‐210‐3p* in EVs from the diabetic, obese group A negative correlation between the *miR‐210‐3p* levels and insulin sensitivity in adipocytes	Serum; ExoQuick ULTRA kit
(Pan et al. 2019)^a^	2019; China	Overweight/obese (*n* = 24) and lean (*n* = 29)	Not mentioned	All female	≥ 23 < 23				Upregulation of *miR‐34a* in both types of adipose tissue from overweight/obese subjects compared to the lean groups Increased TNFα and IL6 in the obese population Lower levels of *miR‐34a* in insulin‐sensitive individuals	Serum; Ultracentrifugation
(Togliatto et al. 2016)	2015; Italy	Obese (*n* = 10) and non‐obese (*n* = 6)	46.6 51.4	4:6 1:5	43.96 ± 4.03 26.6 ± 0.6	FBS (mmol/L) 5.9 ± 2.2 4.8 ± 0.5	Total cholesterol (mg/dL): 97.65 ± 0.93 98 ± 0.55 Triglyceride (mg/dL): 32.6 ± 0.64 26.85 ± 0.42		Downregulation of *miR‐126* in obese derived EVs Obese‐EVs showed impaired angiogenic capabilities Downregulation of *miR‐126*, VEGF, and *MMP‐2* in Obese patients A negative correlation between *miR‐126* and Spred1 content	ASCs NTA NanoSight LM10
(Eirin et al. 2021)	2021; USA	Lean (*n* = 5) and obese (*n* = 5)	24.5 (21–29) 29.3 (24–32)	3:2 (each group)	19.4 (18.4–20.1) 50.1 (40.6–72.6)	FBS (mmol/L) 5 (4.3–5.3) 7.1 (4.6–8.9) HbA1c (mmol/mol): 5.2 (5–5.5) 6.5 (5.2–7.9)	Total cholesterol (mg/dL): 79.3 ± 0.3 93.7 ± 0.9		*has‐miR‐222‐5p*: 1.03 ± 0.27 1.45 ± 0.26 *has‐miR‐888‐5p* 0.96 ± 0.28 1.28 ± 0.33 *hsa‐miR‐6752‐5p*: 1.05 ± 0.42 0.37 ± 0.1 *has‐miR‐6838‐3p*: 5.84 ± 2.62 1.69 ± 1.61 Upregulated miRNAs in Obese‐EVs targeted NFk‐B and MAPK signaling, cytoskeletal organization, and apoptosis Downregulated miRNAs in Obese‐EVs targeted Cell cycle regulation, angiogenesis, Wnt, and MAPK signaling pathways	ASCs NTA Nanosight NS300
(Li et al. 2021)	2021; China	Lean (*n* = 4) and MetS (*n* = 4)	24.5 29.3	2:2 2:2	19.1 ± 0.9 62.2 ± 14.2	FBS (mmol/L): 4.9 ± 0.4 8.2 ± 1.6 HbA1c (%): 5.4 ± 0.2 7.2 ± 0.8^a^	Total cholesterol (mg/dL): 81 ± 0.4 91.9 ± 0.5 Triglyceride (mg/dL): 31.53 ± 0.2 43.6 ± 0.8 HDL (mg/dL) 17.29 ± 0.1 17.47 ± 0.2 LDL (mg/dL) 28.82 ± 0.3 55.86 ± 0.6^a^ ^s^	Systolic BP (mmHg): 113 ± 11.9 150 ± 9.7 Diastolic BP (mmHg): 63.8 ± 5.9 95 ± 4.5 Upregulation of miRNAs that target genes involved in oxidation–reduction Downregulation of miRNAs that influence heart, blood vessel, and skeletal development, transcriptional regulation, apoptosis, and the cell cycle in patients with metabolic syndrome	Upregulated in the MetS group: *miR‐718, −6089, −5089‐3p, −1908‐3p, −665, −7110‐5p, −3185, −4634, −124‐3p, −3178, −4754, −335‐5p, −652‐3p, −3610, −1180‐3p, −3175, −543, −30c‐1‐3p, −363‐3p, −193b‐5p, −2277‐3p, −4691‐3p, −1909‐3p, −3195, −323a‐5p, −1267, −6883‐5p, −5694, −149‐5p, −4453, −1914‐5p, −3908, −5090, −3922‐3p, −6782‐5p, −8052, −2909, −134‐3p, −8064, −4251* Downregulated in the MetS group: *miR‐382‐3p, −3936, −29b‐2‐5p, −6078, −7849‐3p, −4293, −20b‐3p, −6507, −625‐5p, −105‐3p*	Serum/Plasma; exoRNeasy

Abbreviations: ASCs, adipose tissue‐derived stem cells; FABP, fatty acid binding proteins; FBS, fasting blood sugar; GLUT4, glucose transporter type 4; HDL, high‐density lipoprotein; IL‐6, interleukin 6; Klf4, Krüppel‐like factor 4; LDL, low‐density Lipoprotein; MAPK, mitogen‐activated protein kinase; MetS, metabolic syndrome; MMP‐2, matrix metalloproteinase‐2; TGF‐β, tumor growth factor β; TNFa, tumor necrosis factor a; VEGF, vascular endothelial growth factor.

^a^ In vivo studies with further clinical assessment.

Several studies focused on children and adolescents, identifying early obesity‐related molecular alterations; obese children exhibited significantly higher plasma levels of *miR‐33a‐3p* compared with controls (1.33 ± 0.29 vs. 0.58 ± 0.29; *p* = 0.026). This miRNA correlated with insulin resistance and lipid metabolism markers (*p* = 0.01) (Cabiati, Guiducci, et al. 2023). In contrast, reduced levels of *miR‐223‐5p, miR‐33a‐3p, miR‐181a‐5p*, and *miR‐199a‐5p* were reported in obese children in another cohort, indicating heterogeneity in pediatric obesity‐associated miRNA signatures (Cabiati, Randazzo, et al. 2023). In obese adolescents, exosomes derived from visceral and subcutaneous adipose tissue showed upregulation of *miR‐23b* and *miR‐4429*, alongside downregulation of *miR‐148b* and *miR‐4269* (*p* < 0.05). These miRNAs were linked to the TGF‐β and Wnt/β‐catenin pathways. This finding highlighted their involvement in inflammation and adipogenesis (Ferrante et al. 2015).

Distinct miRNA expression patterns were also observed in adult populations; In adult women stratified by BMI, *miR‐320a‐3p, let‐7b‐5p, miR‐106a‐5p*, and *miR‐106b‐5p* were significantly upregulated in obese individuals, while *miR‐186‐3p* and *miR‐146a‐5p* were downregulated (*p* < 0.05) (Santamaria‐Martos et al. 2020). In a comparison between diabetic obese patients and non‐diabetic normal‐weight controls, serum exosomal *miR‐210‐3p* levels were significantly elevated in the diabetic obese group and negatively associated with adipocyte insulin sensitivity (Patra et al. 2024). Moreover, *miR‐34a* was consistently overexpressed in visceral and subcutaneous adipose‐derived exosomes from obese women and positively correlated with circulating TNF‐α and IL‐6 levels. Notably, insulin‐sensitive individuals exhibited lower *miR‐34a* expression. This result suggested a role in obesity‐associated insulin resistance (Pan et al. 2019).

A comparative analysis between lean individuals (BMI = 24.5) and patients with metabolic syndrome (BMI = 29.3) revealed distinct redox‐ and cardiovascular‐related miRNA signatures. For example, redox‐sensitive miRNAs such as *miR‐718*, *miR‐124‐3p*, and *miR‐543* were upregulated in metabolic syndrome patients. Conversely, miRNAs involved in cardiovascular regulation, including *miR‐382‐3p*, *miR‐29b‐2‐5p*, and *miR‐20b‐3p*, were downregulated (Li et al. 2021).

Emerging evidence highlights the contribution of adipose stem cell (ASC)–derived exosomes to obesity‐related vascular impairment; for instance, exosomal *miR‐126* from obese ASCs was associated with impaired angiogenesis, reduced VEGF and MMP‐2 expression, and increased Spred1 levels. This finding suggested a mechanistic link to vascular dysfunction in obesity (Togliatto et al. 2016). In a comparative study of lean and obese adults, ASC‐derived exosomes from obese individuals showed upregulation of *miR‐222‐5p* and *miR‐888‐5p*, targeting NF‐κB/MAPK and cytoskeletal pathways. In contrast, downregulated miRNAs, such as *miR‐6752‐5p* and *miR‐6838‐3p*, were linked to angiogenesis, Wnt/MAPK, and cell‐cycle regulation (Eirin et al. 2021).

#### 3.1.2 MASLD‐Associated Exosomal miRNAs

This systematic review included eleven articles from 2020 to 2024, encompassing studies on MASLD, NASH, obesity, and healthy subjects in China, South Korea, Germany, Egypt, Spain, and Australia (Gim et al. 2021; Zhou et al. 2020; Jiang et al. 2021; Li et al. 2020; Newman et al. 2022; Zhang and Pan 2022; Paluschinski et al. 2023; Liu et al. 2020, 2021; Samy et al. 2024; Infante‐Menéndez et al. 2023). With diverse sex representation, the age of the subjects spanned from children to adults. Exosome isolation was mostly done in serum or plasma; clinical and biochemical data are presented in Table 2.

**TABLE 2:** Summary of clinical and exosomal miRNA findings in NAFLD/MASLD studies.

References	Year and country	Study groups	Age	Female:male	BMI (kg/m^2^)	Glucose profile	Lipid profile	Hepatic profile	Other clinical parameters	Findings	Exosome isolation (source, method)
(Gim et al. 2021)^a^	2021; South Korea	NAFLD (*n* = 41), healthy (*n* = 24), simple steatosis (*n* = 20), NASH (*n* = 19)	55 (43–63)	28:13	29.94 (26.20–33.46)	Not mentioned	Not mentioned	Positive correlation between miRNAs (*miR‐133b* and *miR‐8079*) with ALP and ALT Positive correlation between *miR‐4436a* with AST and GGT Positive correlation between *miR‐7151‐5p* with Total NAS Protective effect of *miR‐133b* on hepatic inflammation Correlation of *let‐7b‐5p* with liver fibrosis‐associated genes	Not mentioned	Not mentioned	Serum; ExoQuick Plus kit
(Zhou et al. 2020)	2020; China	Test set distribution: NAFLD group (*n* = 3) and Control group (*n* = 3) Validation set distribution: NAFLD group (*n* = 17) and Control group (*n* = 17)	11.50 ± 0.3 12.37 ± 0.9 11.42 ± 0.3 11.74 ± 0.6	All male All male 6:11 7:10	30.37 ± 1.0 17.09 ± 0.5 29.09 ± 0.9 17.15 ± 0.2	Not mentioned	Cholesterol (mmol/L): 4.55 ± 0.42 4.29 ± 0.32 4.84 ± 0.22 4.32 ± 0.22 Triglyceride (mmol/L): 1.48 ± 0.06 0.97 ± 0.20 1.67 ± 0.23 0.99 ± 0.14	AST (IU/L): 133.67 ± 32.03 18.67 ± 2.40 95.41 ± 14.51 22.65 ± 0.96 ALT (IU/L): 194.33 ± 25.50 25.67 ± 3.48 161.00 ± 25.5 13.29 ± 1.02	Uric Acid (𝜇mol/L): 448.33 ± 55.03 281.00 ± 31.01 449.06 ± 17.59 303.23 ± 12.24	Upregulation of *miR‐122‐5p, miR‐34a‐5p, miR‐155‐5p*, and *miR‐146b‐3p* in the NAFLD group Correlation between *miR‐122‐5p, miR‐34a‐5p, miR‐155‐5p* and *miR‐146b‐3p* with BMI, ALT, AST and UA	Serum/Plasma; miRCURY Exosome Kit; EM validation
(Jiang et al. 2021)^b^	2021; China	Healthy (*n* = 50) and NAFLD (*n* = 50) (sex‐ and age‐matched)	42.6 ± 1.6 42.2 ± 1.5	14:36 11:39	Not mentioned	Not mentioned	Cholesterol (mmol/L): 4.89 ± 0.12 5.09 ± 0.15 Triglyceride (mmol/L): 1.40 ± 0.11 2.47 ± 0.37 HDL: 1.27 ± 0.03 1.15 ± 0.04 LDL: 3.19 ± 0.10 3.27 ± 0.10 LPa: 210.10 ± 41 126 ± 23.25	AST (IU/L): 22.95 ± 0.84 26.63 ± 1.23 ALP (IU/L): 79.55 ± 2.56 78.59 ± 2.66 GGT (IU/L): 25.60 ± 1.94 50.38 ± 6.10 Bilirubin Total (mg/dL): 13.44 ± 0.6 12.64 ± 0.6 *miR‐122‐5p* and serum ALT levels were significantly increased in NAFLD patients	Uric Acid (𝜇mol/L): 328.60 ± 9.25 373.30 ± 13.46 Urea: 5.36 ± 0.16 5.16 ± 0.17 Serum Creatinine (mg/dL): 69.32 ± 1.5 68.17 ± 1.6 Total Protein: 73.13 ± 0.7 71.71 ± 0.5 LDH (IU/L): 164.50 ± 2.98 175.00 ± 3.47 CK (IU/L): 115.70 ± 13.72 118.20 ± 14.64 Albumin (mg/dL): 45.73 ± 0.4 45.01 ± 0.3 Globulin 27.90 ± 0.4 26.70 ± 0.4 Albumin/Globulin: 1.66 ± 0.03 1.71 ± 0.04	Downregulation of *miR‐135a‐3p, miR‐129b‐5p*, and *miR‐504‐3p* levels in NAFLD patients ROC Curve Analysis: *miR‐135a‐3p*: AUC = 0.849 *miR‐122‐5p*: AUC = 0.790 *miR‐504‐3p*: AUC = 0.708 ALT: AUC = 0.672 Diagnostic Potential: Exosomal *miR‐135a‐3p* was more sensitive than ALT	Not mentioned
(Li et al. 2020)^b^	2020; China	Non‐NAFLD (*n* = 6) and NAFLD (*n* = 6)	Not mentioned	Not mentioned	Not mentioned	Not mentioned	Not mentioned	Not mentioned	Not mentioned	Increased *miR‐199a‐5p* in NAFLD patients Negative correlation between *miR‐199a‐5p* and *MST1* Down‐regulation of Hepatic *MST1* in NAFLD patients *miR‐199a‐5p* contribution to NAFLD development	Not mentioned
(Newman et al. 2022)	2022; Australia	Control (*n* = 14), NAFLD (*n* = 8), NASH (*n* = 6)	46.5 ± 15.7 48.7 ± 17.7 53.2 ± 15.4	8:6 3:8 3:3	Not mentioned	Not mentioned	Not mentioned	Not mentioned	Not mentioned	Increased Levels of *miR‐122, miR‐192*, and *miR‐128‐3p* in NAFLD/NASH patients	Plasma; anti‐ASGR1 IP, Nanosight NS300
(Zhang and Pan 2022)	2021; China	NAFLD (*n* = 5) and Non‐NAFLD with simple obesity (*n* = 5)	13.4 ± 1.3 13.2 ± 0.9	2:3	28.2 ± 3.4 25.9 ± 1.2	Not mentioned	Cholesterol (mmol/L): 5.3 ± 0.3 4.3 ± 0.5 Triglyceride (mmol/L): 1.62 ± 0.2 1.67 ± 0.1 HDL: 1.3 ± 0.3 1.2 ± 0.3 LDL: 2.9 ± 0.2 1.8 ± 0.2	ALT (IU/L): 104.3 ± 3.9 25.9 ± 1.2	Not mentioned	Most important miRNAs with higher expression in NAFLD: *miR‐122‐5p* *miR‐27a*, and *miR‐335‐5p*	Serum exosomes; ExoQuick kit
(Paluschinski et al. 2023)	2023; Germany	NAFLD (*n* = 24), autoimmune hepatitis (AIH; *n* = 9), and healthy donors (*n* = 14)	Not mentioned	Not mentioned	Not mentioned	Not mentioned	Not mentioned	ALP (IU/L): In NAFLD: 75.5 ± 34.6 In AIH: 108.1 ± 32.0 GGT (IU/L): 132.5 ± 156.3 201.6 ± 173.4 Bilirubin Total (mg/dL): 0.55 ± 0.48 0.44 ± 0.1	Albumin (mg/dL): 4.56 ± 0.31 4.29 ± 0.26	Increased *miR‐15b, miR‐16, miR‐26a, miR‐122*, and *miR‐148a* in EVs isolated from AIH and NAFLD patients compared with the healthy group	Serum; NTA Nanosight NS300
(Liu et al. 2020)^b^	2020; China	Control (*n* = 37), NAFL (*n* = 17), and NASH (*n* = 31)	43.8 ± 1.3 37.1 ± 3.1 38.1 ± 2.3	19:18 4:13 9:22	21.4 ± 0.3 26.3 ± 0.91 27.2 ± 0.6	Not mentioned	Cholesterol (mmol/L): 4.5 ± 0.1 4.7 ± 0.2 5.0 ± 0.1 Triglyceride (mmol/L): 0.8 ± 0.1 1.9 ± 0.3 2.2 ± 0.3 HDL: 1.4 ± 0.0 1.2 ± 0.1 1.2 ± 0.1 LDL: 2.4 ± 0.1 2.9 ± 0.2 2.9 ± 0.1	AST (IU/L): 18.8 ± 1.4 30.2 ± 3.8 51.5 ± 6.0 ALT (IU/L): 19.1 ± 2.4 47.8 ± 6.2 80.2 ± 10.4 GGT (IU/L): 19.1 ± 1.9 78.2 ± 36.8 128.2 ± 51.9	Not mentioned	Increased *miR‐192‐5p* in serum and exosomes of NAFLD and NASH patients Increased levels of iNOS, IL‐6, and TNFa (Special M1 macrophages' cytokines) Decreased levels of YM1 in the NAFLD group A negative correlation between *miR‐192‐5p* and Rictor, p‐Akt, and p‐FoxO1 *miR‐192‐5p* role in NAFLD progression	Serum; ExoQuick/ExoQuick‐TC
(Liu et al. 2021)^b^	2021; China	Control (*n* = 40) and NAFLD (*n* = 40)	53.7 ± 10 51.35 ± 9	11:29 9:31	21.96 ± 2.1 28.02 ± 2.1	Not mentioned	Cholesterol (mmol/L): 4.34 ± 0.73 5.43 ± 0.68 Triglyceride (mmol/L): 1.23 ± 0.26 2.21 ± 0.23 HDL: 1.29 ± 0.16 1.98 ± 0.62 LDL: 2.53 ± 0.33 3.76 ± 0.53	AST (IU/L): 19.42 ± 2.92 34.4 ± 6.38 ALT (IU/L): 19.91 ± 2.45 46.37 ± 3.03 GGT (IU/L): 19.58 ± 1.85 77.74 ± 8.93 *miR‐9‐5p* was correlated with hepatocyte ballooning, lobular inflammation, and Steatosis score Higher *miR‐9‐5p* expression levels in patients with higher hepatocellular ballooning;	Not mentioned	Increased *miR‐9‐5p* in EVs from NAFLD and NASH patients Increase in M1 macrophage, iNOS, TNFα, IL‐6, and IL‐1B in NAFLD/NASH group A negative correlation between miR‐9‐5p and TGM2 *miR‐9‐5p* induced macrophage‐mediated inflammation	Serum; Ultracentrifugation
(Samy et al. 2024)	2024; Egypt	Control (*n* = 50) NAFL (*n* = 50), NASH (*n* = 50)	39.18 ± 6.93 41.08 ± 9.15 42.28 ± 8.68	22:28 33:17 27:23	25.316 ± 0.7 32.031 ± 4.1 29.752 ± 5.5	Not mentioned	Total cholesterol 133.64 ± 29.49 212.50 ± 60.87 179.82 ± 45.31 HDL 63.86 ± 3.23 45.27 ± 12.33 42.32 ± 9.88 LDL 88.34 ± 14.54 129.19 ± 50.23 165.20 ± 8.40 Triglycerides 83.36 ± 15.26 180.00 ± 104.05 234.48 ± 94.70	AST: 18.38 ± 4.23 39.06 ± 24.68 57.90 ± 36.02 ALT: 16.34 ± 5.20 38.48 ± 24.14 62.76 ± 30.67 ALP: 43.96 ± 23.16 111.14 ± 69.40 115.70 ± 58.82 Total‐bilirubin: 0.62 ± 0.21 0.78 ± 0.26 0.78 ± 0.31	SBP (mmHg): 111.40 ± 9.04 116.90 ± 8.97 118.20 ± 10.49 DBP (mmHg): 70.60 ± 7.93 70.30 ± 7.03 76.20 ± 7.80 Albumin: 4.33 ± 0.50 4.35 ± 0.45 4.18 ± 0.47 LPS: 1.76 ± 0.35 2.26 ± 1.36 4.12 ± 0.77 TLR‐4: 1.32 ± 0.41 2.23 ± 0.90 2.65 ± 1.00 FoxO3: 0.69 ± 0.20 1.47 ± 0.68 2.59 ± 0.85 PPAR‐γ: 0.40 ± 0.11 0.81 ± 0.44 1.66 ± 0.79 Adiponectin: 216.15 ± 43.3 117.54 ± 84.8 42.96 ± 19.58	‐ *miR‐122* ↑, *miR‐298* ↓, *miR‐342* ↓ in NAFL and NASH—*miR‐200* ↓ and *miR‐128* ↑ in NASH—ROC (NAFLD diagnosis): *miRNA‐122 (*cut‐off = 1.24, AUC = 0.67, 68% sensitivity, and 70% specificity) *miRNA‐128* (cut‐off = 1.20, AUC = 0.76, 76% sensitivity, and 80% specificity) *miRNA‐200* (cut‐off = 0.89, AUC = 0.96, 88% sensitivity, and 94% specificity) *miRNA‐298* (cut‐off = 0.77, AUC = 0.98, 90% sensitivity, and 100% specificity) *miRNA‐342* (cutoff = 0.99, AUC = 0.94, 92% sensitivity, and 90% specificity)	Blood; Ultracentrifugation
(Infante‐Menéndez et al. 2023)^b^	2023; Spain	Normal Liver (NL) (*n* = 21) and NAFL (*n* = 30)	48.33 ± 16 54.13 ± 13	14:7 17:13	24.73 (22.34–30.28) 27.65 (25.87–33.84)	Glucose (mg/dL): 95.00 (87.50–108.00) 97.00 (92.75–107.50) Insulin (μU/mL): 7.4 (5.3–8.6) 9.3 (7.4–13.38) HOMA‐IR: 1.6 (1.10–2.18) 2.70 (1.78–3.37)	TG: 87 (66.50–106) 123.4 (101–155.3) HDL‐Ch: 50 (46–65.50) 45 (39.75–53.75)	ALT: 14 (12–23.50) 19.50 (14.75–29.25) AST: 17 (14.50–19) 17 (15–24.25) Steatosis grade: NL in Grade 0 NAFL in Grade 1–3 Inflammation grade: NL in Grade 0 NAFL in Grade 0–1 Ballooning grade: NL in Grade 0 NAFL in Grade 0 (*n* = 30) NAS score: NL in Grade 0 NAFL in Grades 1–3	Not mentioned	‐ *let‐7d‐5p* ↓ in NAFL—ROC (NAFL diagnosis): *Let‐7d‐5p* (AUC = 0.8571, Cut‐off: ≤ 0.6461, 87.5% sensitivity, and 85.71% specificity)	Plasma; Total Exosome Isolation Kit (Invitrogen)

Abbreviations: AIH, autoimmune hepatitis; ALP, alkaline phosphatase; ALT, alanine transaminase; AST, aspartate transaminase; DBP, diastolic blood pressure; FoxO3, Forkhead box protein O3; GGT, gamma‐glutamyltransferase; IL‐6, interleukin 6; Inos, inducible nitric oxide synthase; LDH, lactate dehydrogenase; LPS, lipopolysaccharide; MASLD, metabolic dysfunction associated steatotic liver disease; NAFLD, non‐alcoholic fatty liver disease; NASH, non‐alcoholic steatohepatitis; PPAR‐γ, peroxisome proliferator‐activated receptor γ; SBP, systolic blood pressure; TG, triglycerides; TLR‐4, toll‐like receptor; TNFα, tumor necrosis factor a; Total NAS, total NAFLD activity score; UA, uric acid.

^a^ Characteristics are related to the NAFLD group.

^b^ In vivo studies with further clinical assessment.

Several studies reported significant associations between specific exosomal miRNAs and liver enzymes or metabolic parameters; for instance, *miR‐133b*, *miR‐8079*, and *miR‐4436a* were positively correlated with ALP, ALT, AST, and GGT. Interestingly, *miR‐133b* also demonstrated anti‐inflammatory activity (Gim et al. 2021). Moreover, *miR‐7151‐5p* was correlated with the non‐alcoholic fatty liver disease (NAFLD) Activity Score (NAS), and *let‐7b‐5p* was associated with fibrosis severity (Gim et al. 2021). *MiR‐122‐5p*, *miR‐34a‐5p*, *miR‐155‐5p*, and *miR‐146b‐3p* were elevated in MASLD and correlated with BMI, ALT, AST, and uric acid levels (Zhou et al. 2020). These findings suggest that exosomal miRNAs reflect both liver injury and metabolic status in MASLD patients.

Several miRNAs demonstrated differential expression between MASLD/NASH patients and controls with simple obesity; for example, upregulated miRNAs in MASLD included *miR‐122‐5p*, *miR‐27a*, *miR‐335‐5p*, *miR‐192*, *miR‐128‐3p*, *miR‐9‐5p*, *miR‐15b*, *miR‐16*, *miR‐26a*, and *miR‐148a* (Newman et al. 2022; Zhang and Pan 2022; Paluschinski et al. 2023; Liu et al. 2021). In contrast, downregulated miRNAs in MASLD included *miR‐135a‐3p, miR‐129b‐5p, miR‐504‐3p, miR‐298, miR‐342*, and *let‐7d‐5p* (Zhou et al. 2020; Liu et al. 2021; Samy et al. 2024).

Several miRNAs showed diagnostic potential. Receiver Operating Characteristic (ROC) analysis indicated *miR‐135a‐3p* had superior diagnostic accuracy compared to ALT (Jiang et al. 2021). Moreover, *miR‐298* (AUC = 0.98), *miR‐200* (AUC = 0.96), and *miR‐342* (AUC = 0.94) distinguished MASLD from controls with high sensitivity and specificity (Samy et al. 2024). Plasma exosomal *let‐7d‐5p* distinguished MASLD from normal liver histology with AUC = 0.8571, 87.5% sensitivity, and 85.71% specificity (cut‐off ≤ 0.6461) (Infante‐Menéndez et al. 2023). These results underscore the potential of specific exosomal miRNAs as non‐invasive biomarkers for MASLD diagnosis and early detection.

Several studies highlighted the role of exosomal miRNAs in mediating inflammation and disease progression; for example, higher levels of *miR‐199a‐5p* in MASLD were negatively correlated with macrophage‐stimulating 1 (MST1). This mechanism may be involved in inflammation‐driven MASLD pathogenesis (Li et al. 2020). Moreover, higher *miR‐192‐5p* levels in MASLD and NASH were also correlated with elevated pro‐inflammatory mediators (iNOS, IL‐6, TNFα) and negatively correlated with Rictor, p‐Akt, and p‐FoxO1 signaling proteins (Liu et al. 2020). Finally, highly expressed *miR‐9‐5p* in MASLD/NASH extracellular vesicles was correlated with hepatocyte ballooning, lobular inflammation, and steatosis scores. This miRNA was also linked to increased M1 macrophage activation and inflammatory cytokine production, with negative correlation to TGM2 expression (Liu et al. 2021).

By conducting comparative analysis across studies, *miR‐122‐5p, miR‐27a*, and *miR‐335‐5p* were key discriminators between MASLD and simple obesity (Zhang and Pan 2022). Moreover, higher levels of miR‐122, miR‐192, and miR‐128‐3p were reported in MASLD/NASH compared to healthy controls (Newman et al. 2022). Finally, there were higher levels of *miR‐15b, miR‐16, miR‐26a, miR‐122*, and *miR‐148a* in MASLD and autoimmune hepatitis compared with healthy donors (Paluschinski et al. 2023).

### 3.2 Bioinformatics Analysis Findings

#### 3.2.1 Common miRNAs in Obesity and MASLD

Literature review identified 93 obesity‐ and 24 MASLD‐associated miRNAs, with *let‐7b* and *miR‐335‐5p* overlapping. Based on miRWalk database, we selected the 3UTR and binding *p* > 0.95 and 2526 common miRNA target (see Supporting Information). To increase the reliability of the predictions, the TargetScan database was used and filtering was applied based on strong binding site types, including 8mer and 7mer sites. Furthermore, experimentally validated miRNA‐target interactions were investigated using the miRTarBase database. Only interactions supported by strong experimental evidence were considered, including luciferase reporter assay, qPCR, Western blot, and CLIP experiments. This approach allowed us to incorporate evidence not only at the RNA level but also at the protein level. The results indicated that both *let‐7b* and *miR‐355‐5p* had credible predicted interactions, and a subset of their target genes had also been experimentally validated. These findings further strengthened the reliability of the bioinformatics analysis. The detailed results have been provided in Supporting Information.

#### 3.2.2 Functional Enrichment Results

**FIGURE 3:**
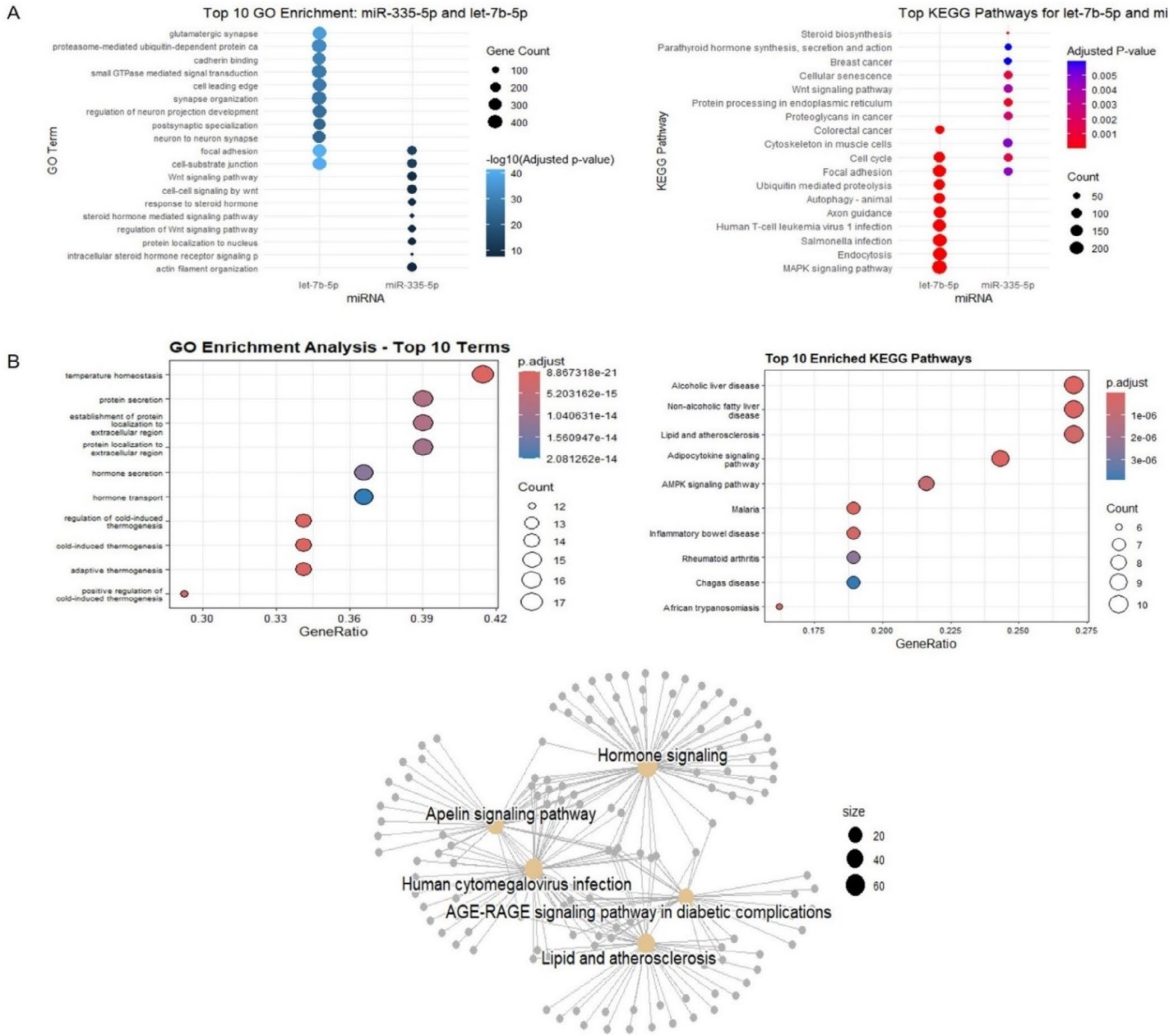
Dot plots for the ten most enriched terms from GO and KEGG enrichment analysis based on two approaches: (A) Target genes of *let‐7b* and *miR‐335‐5p*, and (B) 40,410 common mRNA targets from miRWalk.

Figure 3 illustrates enrichment results for two datasets: (A) *let‐7b* and *miR‐335‐5p*, and (B) 2526 common target genes from miRWalk. For the first dataset, GO terms were enriched in biological processes like steroid hormone response, protein localization, Wnt signaling, cell adhesion, and cytokine activity. Cellular components affected focal and cell‐substrate adhesion, while molecular functions comprised cytokine receptor binding and sterol transporter activity. KEGG analysis picked out steroid biosynthesis, ER protein processing, cell cycle regulation, cancer pathways (e.g., breast cancer), Wnt signaling, and focal adhesion.

For the second data set, GO terms identified roles in temperature homeostasis, secretion of proteins, and extracellular protein localization. KEGG analysis showed highly significant enrichment for adipocytokine signaling (p.adjust = 1.72 × 10^−9^), MASLD and ALD pathways, and several immune‐related pathways (e.g., IBD, RA, malaria, cytokine interactions). Key signaling pathways were AMP‐activated protein kinase (AMPK), JAK–STAT, and FoxO. Metabolic and endocrine dysfunction, such as insulin resistance and diabetic complications, were prominent. Infection‐related processes (e.g., TB, influenza, HBV, COVID‐19) and aging‐related processes (e.g., longevity regulation, necroptosis) were also enriched.

#### 3.2.3 miRNA–mRNA Network Construction

**FIGURE 4:**
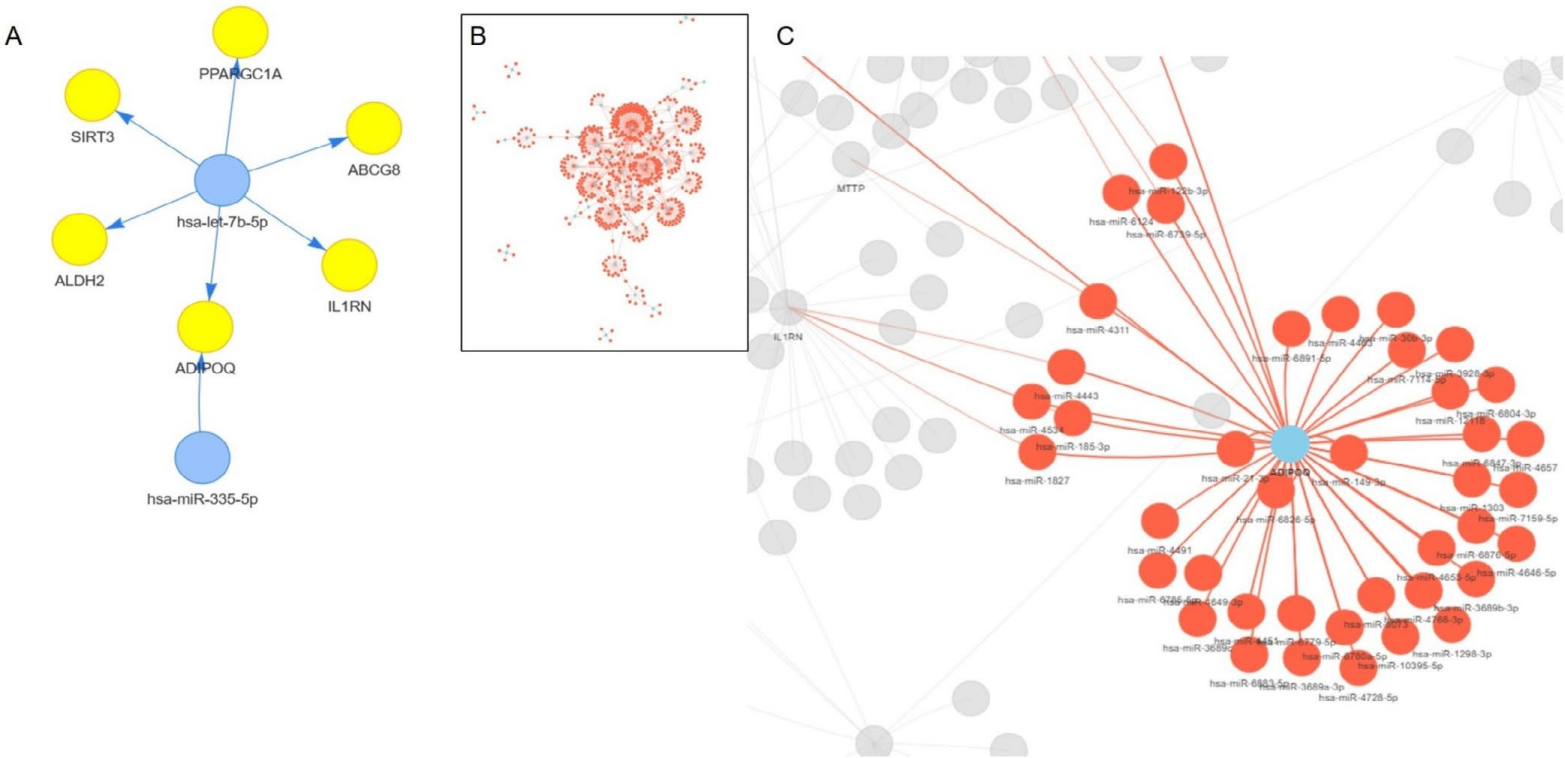
miRNA–mRNA network construction for significant miRNAs identified in (A) systematic review literature (*let‐7b* and *miR‐335‐5p*) and (B) the miRWalk database. (C) Schematic illustration of the miRNA–mRNA network with a focus on *ADIPOQ* mRNA as a target gene of both *let‐7b* and *miR‐335‐5p* within the miRWalk‐based network.

Figure 4A illustrates the interaction network of *let‐7b* and *miR‐335‐5p*, with node size reflecting degree centrality. Figure 4B depicts a broader regulatory network of 2393 miRNAs and 42 target genes from the miRWalk analysis (see details in the Supporting Information). Central hubs included *hsa‐miR‐6085* and *hsa‐miR‐6753‐5p*, linked to genes involved in metabolism and inflammation. Figure 4C highlights the subnetwork centered on *ADIPOQ*, a shared target of *let‐7b* and *miR‐335‐5p*, using the network obtained from the miRWalk analysis.

## 4 Discussion

The present study integrates evidence from a systematic review with large‐scale bioinformatics prediction to clarify shared miRNA‐mediated mechanisms linking obesity and MASLD. Three key messages emerge: First, despite the complexity suggested by computational analyses, only a limited number of miRNAs, most notably *let‐7b* and *miR‐335‐5p*, are consistently supported by experimental evidence in both conditions. Second, these miRNAs converge on metabolic‐inflammatory pathways, particularly those regulating adiponectin signaling, insulin sensitivity, and macrophage polarization. Third, while bioinformatics approaches expand the regulatory landscape substantially, they primarily generate hypotheses that require experimental validation before clinical translation.

### 4.1 Experimentally Supported miRNAs Shared by Obesity and MASLD

Based on the systematic review, *let‐7b* and *miR‐335‐5p* were the only miRNAs reproducibly implicated in both obesity and MASLD across independent studies. This restricted overlap likely reflects both publication bias and the limited availability of validated exosomal miRNA datasets, rather than a lack of biological relevance of other miRNAs. Also, missing functional assays in humans are a strong limitation. In mouse studies, it is easier to analyze the target organ, tissue of origin, and circulating miRNAs simultaneously. Some miRNA‐mRNA interactions are conserved, as well as some miRNAs are conserved between human and mouse. The use of (human) immortalized or cancer cell lines is a limitation for the prediction and validation due to their limited similarity to the physiological state. Therefore, the bioinformatic approach is highly theoretical and predictive. The here presented results should be considered with extreme caution.

*Let‐7b*, as a conserved miRNA, has been experimentally linked to hepatic fibrosis, fatty acid transport, and adipose tissue remodeling through modulation of the TGF‐β signaling pathway, including inhibition of white‐to‐beige adipocyte transdifferentiation (Santamaria‐Martos et al. 2020; Gim et al. 2021). *MiR‐335‐5p*, previously associated with insulin signaling, Wnt pathways, and metabolic dysregulation, is elevated in both obesity and MASLD and appears to exert pleiotropic effects on lipid metabolism and inflammation (Li et al. 2021; Zhang and Pan 2022; Ye et al. 2021). The convergence of independent experimental studies on these two miRNAs suggests that they represent core regulatory nodes rather than peripheral signals. Nevertheless, their biological roles appear to be modulated by age, disease stage, and tissue context, as discussed below.

### 4.2 Age Dependence

A clear age‐dependent pattern emerges across both MASLD and obesity studies, indicating that exosomal miRNA signatures evolve with disease stage and developmental context.

In MASLD, pediatric cohorts (mean age: 11–14 years) predominantly exhibited exosomal miRNAs linked to hepatic steatosis and metabolic burden, rather than fibrosis or advanced inflammation. Studies by Zhou et al. (2020) and Zhang and Pan (2022) consistently reported upregulation of *miR‐122‐5p, miR‐34a‐5p*, and *miR‐335‐5p* in children with MASLD (Zhang and Pan 2022). These miRNAs are well‐established regulators of lipid metabolism and hepatocellular stress, supporting the interpretation that pediatric MASLD is largely driven by reversible metabolic overload rather than irreversible liver injury. In contrast, adult MASLD cohorts (mean age: 40–55 years) displayed exosomal miRNA profiles increasingly reflective of inflammatory activation, immune remodeling, and disease progression. Liu et al. (2020, 2021) identified *miR‐192‐5p* and *miR‐9‐5p* as key mediators of M1 macrophage polarization, correlating with lobular inflammation, hepatocyte ballooning, and elevated pro‐inflammatory cytokines. Similarly, Samy et al. (2024) demonstrated that *miR‐298, miR‐200*, and *miR‐342* showed excellent diagnostic performance in distinguishing MASLD from NASH, consistent with their association with more advanced inflammatory phenotypes. Collectively, these findings support a progressive shift from metabolic to inflammatory and fibrotic miRNA signatures with advancing age and disease severity.

A comparable age‐related trend was observed in obesity‐related studies. In children and adolescents, exosomal miRNAs were primarily associated with early metabolic dysregulation and adipose tissue remodeling, rather than chronic inflammation. For example, Cabiati, Guiducci, et al. (2023) and Cabiati, Randazzo, et al. (2023) reported altered *miR‐33a‐3p* levels in obese adolescents (mean age: 13 years), which correlated strongly with insulin resistance and lipid abnormalities despite relatively preserved blood pressure and glycemic control. Ferrante et al. (2015) further identified miRNAs involved in TGF‐β and Wnt/β‐catenin signaling, as pathways central to adipogenesis and tissue plasticity during growth. This highlights the developmental nature of miRNA regulation in pediatric obesity (Ferrante et al. 2015). By contrast, adult obesity studies consistently demonstrated miRNA signatures linked to chronic inflammation, vascular dysfunction, and insulin resistance. Santamaria‐Martos et al. (2020) showed progressive upregulation of *let‐7b‐5p* and *miR‐320a‐3p* with increasing BMI in adult women, accompanied by worsening HOMA‐IR and lipid profiles. Additionally, Togliatto et al. (2016) and Eirin et al. (2021) reported downregulation of *miR‐126* and other angiogenesis‐related miRNAs in adipose‐derived exosomes from obese adults. This implicates impaired vascular repair and endothelial dysfunction (Togliatto et al. 2016; Eirin et al. 2021). Together, these data indicate a transition from metabolic programming in youth to vascular and inflammatory pathology in adulthood, reflected in distinct exosomal miRNA profiles.

Despite these age‐related differences, certain miRNAs, notably members of the *let‐7* family and *miR‐335‐5p*, were detected in both pediatric and adult populations (Santamaria‐Martos et al. 2020; Gim et al. 2021; Zhang and Pan 2022; Infante‐Menéndez et al. 2023). This suggests that these miRNAs may function as core regulators across the lifespan, while their downstream targets and biological effects vary according to developmental stage, metabolic context, and disease duration. However, the absence of age‐stratified analyses within most studies precludes definitive conclusions regarding age‐specific functionality. This limitation underscores the need for explicit age‐stratified and longitudinal investigations to clarify whether shared miRNAs exert qualitatively distinct effects in children and adults.

### 4.3 Inflammation as a Shared Mechanism

Inflammation represents the most robust mechanistic bridge between obesity and MASLD, supported by multiple experimental studies (Jiang et al. 2021; Zhang and Pan 2022; Paluschinski et al. 2023; Liu et al. 2020; EASL, EASD, and EASO 2024). Several exosomal miRNAs have been shown to actively shape macrophage polarization and inflammatory tone.

For example, *miR‐192‐5p*, upregulated in serum exosomes from MASLD patients, was inversely correlated with Rictor, p‐Akt, and p‐FoxO1, favoring polarization toward the pro‐inflammatory M1 macrophage phenotype (Liu et al. 2020). Similarly, increased serum *miR‐9‐5p* expression was associated with reduced TGM2, a marker of anti‐inflammatory M2 macrophages, alongside elevated iNOS, TNFα, IL‐6, and IL‐1β levels (Liu et al. 2021). Other miRNAs, including serum *miR‐34a*, were shown to suppress Klf4 expression, further inhibiting M2 polarization and perpetuating inflammation (Pan et al. 2019; Zhou et al. 2020). In parallel, plasma *miR‐199a‐5p* suppressed MST1, promoting hepatic lipogenesis and metabolic dysregulation via AMPK and SREBP‐1c pathways (Cabiati, Guiducci, et al. 2023; Cabiati, Randazzo, et al. 2023; Li et al. 2020). These experimentally validated findings support a model in which circulatory exosomal miRNAs act as intercellular amplifiers of metabolic inflammation, linking adipose tissue dysfunction to hepatic injury.

### 4.4 Insights From Bioinformatics: Hypothesis‐Generating Networks

In contrast to the relatively small number of experimentally validated miRNAs, miRWalk‐based prediction identified tens of thousands of shared miRNAs and an extensive network of potential target genes. While this discrepancy highlights the exploratory nature of computational analyses, it also underscores the complexity of miRNA‐mediated regulation.

Functional enrichment of predicted targets revealed involvement in AMPK, FoxO, JAK–STAT, MAPK, and endocytosis pathways, as well as immune and infection‐related signaling. These findings are consistent with the systemic and immune‐metabolic nature of obesity and MASLD but should be interpreted cautiously. Importantly, bioinformatics predictions do not imply functional relevance without experimental confirmation.

Supporting this distinction, experimental work by Eirin et al. (2021) demonstrated that adipose stem cell‐derived exosomal miRNAs differ markedly between lean and obese individuals, with overexpressed miRNAs (e.g., *miR‐222‐5p*, *miR‐888*‐5p) targeting NF‐κB/MAPK signaling and downregulated miRNAs affecting angiogenesis and cell‐cycle regulation. These results align with, but do not validate, the broader computational predictions.

### 4.5 ADIPOQ as a Central Integrator of Metabolic Signals

Network analysis identified *ADIPOQ* as a hub gene regulated by *let‐7b* and *miR‐335‐5p*, positioning adiponectin signaling at the intersection of obesity and MASLD. Adiponectin is a well‐established mediator of insulin sensitivity, lipid oxidation, and anti‐inflammatory responses via AMPK and PPARα activation (Roy and Palaniyandi 2021; Wang et al. 2022; Engin 2024; Hoffstedt et al. 2004; Gavrila et al. 2003; Alsaedi et al. 2025). Higher adiponectin levels are consistently associated with reduced adiposity and improved metabolic outcomes (Wang et al. 2022; Alsaedi et al. 2025).

Although our analysis highlights *ADIPOQ* as a central regulatory node, direct experimental evidence linking miRNA‐driven regulation of *ADIPOQ* to disease progression in humans is still limited. Validation of the *let‐7b–miR‐335‐5p–ADIPOQ* axis in cellular and animal models therefore represents a critical next step.

### 4.6 Dietary and Nutritional Modulation of Exosomal miRNAs

Our findings have practical implications for the design of dietary strategies and functional food products targeting individuals with obesity or MASLD. By integrating experimentally validated miRNA‐target interactions with bioinformatics predictions, our analysis highlights metabolism‐responsive miRNAs, particularly *let‐7b* and *miR‐335‐5p*, and *ADIPOQ*‐driven pathways as potential molecular links between diet and metabolic health. These miRNAs may serve not only as biomarkers of dietary response but also as mechanistic mediators through which specific foods or nutrients exert beneficial effects.

*Let‐7b* emerges as a key candidate for diet‐based modulation. Evidence from human intervention studies suggests that its expression responds to changes in energy balance and macronutrient composition, including caloric restriction, fasting, and ketogenic diets (Jayasooriya et al. 2022; Lilja et al. 2021; Cannataro et al. 2019; Tutino et al. 2021). In the context of obesity and MASLD, this metabolic sensitivity is highly relevant, as *let‐7* family members are known to regulate genes involved in insulin signaling, lipid metabolism, inflammation, and adipocyte function (Santamaria‐Martos et al. 2020; Gim et al. 2021; Infante‐Menéndez et al. 2023). Diets that improve insulin sensitivity and reduce hepatic fat accumulation, such as controlled calorie restriction, low‐carbohydrate ketogenic approaches, or intermittent fasting, may exert their benefits in part through modulation of pathways regulated by *let‐7b* (Jayasooriya et al. 2022; Lilja et al. 2021; Cannataro et al. 2019; Tutino et al. 2021). However, the direction of *let‐7b* modulation appears to depend on metabolic context and tissue source; for example, a 6‐week weight loss intervention combining moderate calorie restriction and exercise in overweight adults resulted in significant reductions in circulating *hsa‐let‐7b* levels (Jayasooriya et al. 2022). In contrast, short‐term fasting increased *let‐7b‐5p* expression in stool samples from healthy adults (Lilja et al. 2021), and a ketogenic diet upregulated *let‐7b‐5p* in the circulation after 6 weeks (Cannataro et al. 2019). Beyond global dietary patterns, specific food‐derived bioactive compounds have also been implicated in the regulation of *let‐7b*. Catechins, particularly epigallocatechin gallate (EGCG), have been shown to increase *miR‐let‐7b* expression in in vitro and in vivo melanoma models, leading to tumor growth suppression through modulation of cAMP/PKA/PP2A signaling pathways (Yamada et al. 2016). While these data originate from cancer models, they provide proof of principle that defined dietary polyphenols can directly influence miRNA expression. Whether similar mechanisms operate in metabolic tissues remains largely unknown.

Evidence linking diet to *miR‐335‐5p* is more limited. Notably, a randomized controlled trial investigating high fresh grape consumption in overweight adults reported downregulation of circulating *miR‐335* after a 21‐day intervention (Tutino et al. 2021). Given that *miR‐335‐5p* emerged as a relevant node in our integrative analysis, this observation suggests that polyphenol‐rich foods may influence this miRNA and potentially affect adipogenesis, insulin signaling, or inflammatory pathways. However, data are sparse and mechanistic studies directly linking dietary components to *miR‐335‐5p* regulation are currently lacking.

In the case of *ADIPOQ*‐related pathways, clinical trials suggest that hypocaloric diets enriched in monounsaturated or polyunsaturated fatty acids can differentially affect adiponectin concentrations depending on *ADIPOQ* genetic variants, particularly *rs266729*. Individuals with the CC genotype show greater improvements in adiponectin levels and metabolic parameters after weight loss, regardless of whether their diet is rich in monounsaturated or polyunsaturated fats (de Luis et al. 2020). These findings highlight the complex interplay between diet, genetics, and adipokine regulation (de Luis et al. 2020; Rohde et al. 2015; Błażejewska et al. 2025). Although our study identifies *ADIPOQ* as a major target in miRNA regulatory networks, direct evidence linking diet‐induced changes in specific miRNAs to *ADIPOQ* expression in humans is still lacking. This represents a clear knowledge gap.

## 5 Conclusion

By integrating experimentally validated evidence with computational prediction, this study delineates both high‐confidence regulatory mechanisms and broader hypothesis‐generating networks linking obesity and MASLD. The systematic review identifies *let‐7b* and *miR‐335‐5p* as reproducible and biologically relevant miRNAs shared across both conditions, while bioinformatics analyses extend this framework and highlight *ADIPOQ*‐centered signaling pathways as potential points of molecular convergence.

At the same time, these conclusions should be interpreted in light of several limitations. The number of experimentally validated studies remains limited; the included cohorts are heterogeneous with respect to comorbidities, age, gender, ethnicity, disease severity, and clinical characteristics. A major limitation of the current evidence base is the lack of age‐stratified analyses, despite the inclusion of both pediatric and adult populations. The reviewed data suggest that exosomal miRNA profiles differ substantially between children and adults, reflecting distinct stages of metabolic dysfunction and liver disease progression. Future studies should therefore incorporate explicit age‐based stratification and longitudinal designs to determine whether shared miRNAs act through conserved or age‐specific mechanisms. Such efforts will be essential for translating exosomal miRNA research into clinically meaningful, age‐appropriate diagnostic and therapeutic strategies for obesity‐associated MASLD. In addition, many of the inferred pathways are based on computational prediction and require functional validation.

The primary aim of our study was to evaluate exosomal miRNAs in obesity and MASLD regardless of their tissue origin; our analysis focused on the overall profile of reported exosomal/circulating miRNAs. Among the extracted studies, only three investigated EV miRNAs derived directly from adipose tissue (Ferrante et al. 2015; Togliatto et al. 2016; Eirin et al. 2021), whereas the majority assessed circulating miRNAs without identifying their tissue of origin. Therefore, the exact source of the reported circulating miRNAs was not to be definitively determined. This limitation restricted the ability to establish a direct mechanistic link between adipose tissue‐derived EV miRNAs and liver signaling pathways in obesity and MASLD. Future studies should aim to identify the organ‐specific origin of the circulating miRNAs as well as the target tissues of the adipose tissue–derived EV miRNAs that show differential expression under these conditions, in order to better clarify adipose‐liver inter‐organ communication. Moreover, another limitation was the methodological heterogeneity among the included studies, including differences in miRNA profiling techniques, normalization approaches, and biological sample sources, which may affect the direct comparability of reported exosomal miRNA profiles.

Taken together, these findings provide a conceptual roadmap for future research rather than immediate clinical application. Focused experimental studies, ideally incorporating age‐specific and longitudinal designs, are needed to validate the functional roles of *let‐7b, miR‐335‐5p*, and *ADIPOQ*‐related networks. In the longer term, such efforts may support the development of miRNA‐based biomarkers or therapeutic strategies for obesity‐associated MASLD, but substantial mechanistic and translational work remains necessary before clinical implementation.

## Author Contributions

**Qigui Mo:** conceptualization, visualization, writing – original draft. **Mahdieh Soleimani:** formal analysis, software. **Reza Afrisham:** conceptualization, writing – review and editing, supervision. **Masoomeh Hamdi:** writing – original draft, methodology. **Arian Alidadipour:** writing – original draft, methodology. **Xiaolei Miao:** conceptualization, writing – review and editing, supervision. **Molood Bagherieh:** writing – review and editing, supervision. **Maryam Davoudi:** methodology, writing – review and editing. **Amirreza Ghafourian:** writing – original draft, data curation, methodology.

## Funding

This project was supported by the Doctoral Research Fund of Hubei University of Science and Technology (Grant number: BK202332; 2022TNB06). In addition, this work was supported by the Doctoral Research Fund of Hubei University of Science and Technology (Grant number: Q201810), Hubei Natural Science Foundation (2026AFC0525), and Scientific Research Program of the Hubei Provincial Department of Education (Q20242808). Moreover, this project was also supported by Mazandaran University of Medical Sciences (Grant number: 24058).

## Ethics Statement

Ethical approval was granted by the Research Ethics Committee of Mazandaran University of Medical Sciences (IR.MAZUMS.RIB.REC.1404.010).

## Consent

The authors have nothing to report.

## Conflicts of Interest

The authors declare no conflicts of interest.

## Supporting information


**Data S1:** fsn371901‐sup‐0001‐Supinfo.rar.

## Data Availability

The data that supports the findings of this study are available in the Supporting Information of this article.
